# Seasonal variation in the incidence of preeclampsia and eclampsia in tropical climatic conditions

**DOI:** 10.1186/1472-6874-7-18

**Published:** 2007-10-15

**Authors:** Vidya Subramaniam

**Affiliations:** 1 Department of Obstetrics and Gynaecology, Bassetlaw Hospital, Blyth Road, Worksop, Nottinghamshire, UK

## Abstract

**Background:**

Observational studies have demonstrated various correlations between hypertensive disorders of pregnancy and different weather parameters. We aim to study if a correlation exists between the incidence of eclampsia and pre-eclampsia and various weather parameters in the tropical coastal city of Mumbai which has the distinction of having relatively uniform meteorological variables all throughout the year, except for the monsoon season.

**Methods:**

We retrospectively analysed data from a large maternity centre in Mumbai, India over a period of 36 months from March 1993 to February 1996, recording the incidence of preeclampsia and eclampsia. Meteorological data was acquired from the regional meteorological centre recording the monthly average temperature, humidity, barometric pressure and rainfall during the study period. Study period was then divided into two climate conditions: monsoon season (June to August) and dry season September to May. The incidence of preeclampsia and eclampsia and the meteorological differences between the two seasons were compared.

**Results:**

Over a 36-month period, a total of 29562 deliveries were recorded, of which 1238 patients developed preeclampsia (4.18%) and 34 developed eclampsia (0.11%). The incidence of preeclampsia did not differ between the monsoon and the dry season (4.3% vs. 4.15%, p = 0.5). The incidence of eclampsia was significantly higher in the monsoon (0.2% vs. 0.08%, p = 0.01). The monsoon was significantly cooler (median maximum temperature 30.7°C vs. 32.3°C, p = 0.01), more humid (median relative humidity 85% vs. 70%, p = 0.0008), and received higher rainfall (median 504.9 mm vs. 0.3 mm, p = 0.0002) than the rest of the year. The median barometric pressure (1005 mb) during the monsoon season was significantly lower than the rest of the year (1012 mb, p < 0.0001).

**Conclusion:**

In the tropical climate of Mumbai, the incidence of eclampsia is significantly higher in monsoon, when the weather is cooler and humid with a lower barometric pressure than the rest of the year. This effect is not seen with preeclampsia. This strengthens the association of low temperature and high humidity with triggering of eclampsia.

## Background

The aetiology of preeclampsia and eclampsia is not fully understood. Previous studies have shown a variable association of preeclampsia and eclampsia with the changing weather patterns of different seasons. These association studies often compared the incidence of preeclampsia and eclampsia against the climatic patterns seen in the three or four characteristic seasons in the study area. Studies coming from different parts of the world frequently give opposing results. There are two studies which demonstrate no relationship of meteorological factors on the incidence of eclampsia [1,2]. Most data however tends to suggest that eclampsia is associated with cooler temperatures or winter or with increased humidity or rainfall [3-5]. On the other hand, Griswold et al in their study from Florida, USA suggest higher incidence of eclampsia in the hurricane weather, which is characterised by higher temperatures rather than lower, increased humidity and reduced barometric pressures [6]. Available studies on the association of preeclampsia with various weather patterns are also divided in their conclusions. Majority of published studies conclude that preeclampsia occurs more frequently in winter [7-10]. Conversely, Tan et al have suggested that preeclampsia is common in summer [11]. Wacker and colleagues found no statistically different frequency of preeclampsia in the dry and wet seasons that occur in Zimbabwe [12]. All these studies have assessed if there was a seasonal variation in the incidence of preeclampsia. Interestingly Phillips et al, in their study, have evaluated the link between the timing of conception with risk of preeclampsia [13]. They found the highest risk of preeclampsia in conceptions occurring in the summer season, whereas there was no significant variation in the incidence of preeclampsia based on the timing of delivery.

In our study, we intend to establish if any such association exists in the distinct climate of the tropical coastal city of Mumbai, India. Mumbai has a remarkably stable climate all throughout the year, except for the monsoon season, where the weather pattern is overwhelmed by heavy rainfall. We aim to assess the association of preeclampsia and eclampsia with various weather parameters in this unique climatic condition.

## Methods

The study population included all patients delivered in a large maternity centre in Mumbai. The project did not entail accessing any individual patient data or identifiable information and was reviewed by the hospital research committee. Prospectively maintained database recording all deliveries and associated complications was analysed retrospectively for a three-year period from March 1993 to February 1996 to identify the total number of deliveries, total number of preeclampsia cases and the total number of eclampsia cases. The data was then divided into two groups based on the two main seasons (June to August as monsoon season and September to May as the dry season). This division was based on the fact that the coastal city of Mumbai experiences a remarkably stable weather pattern all throughout the year except during the monsoon season. Monthly averages of meteorological parameters (maximum daily temperature, morning humidity, daily rainfall and morning barometric pressure) were acquired from the Regional Meteorological Centre, Mumbai for the study period.

The incidence of preeclampsia and eclampsia and the meteorological parameters between the two seasons (June to August and rest of the year) were compared. Data was compared using Fisher's exact test, Chi-square test and Mann-Whitney test using GraphPad Prism version 4.03 for windows, GraphPad Software, San Diego California, USA.

## Results

Over a 36-month period, a total of 29562 deliveries were recorded, of which 1238 patients developed preeclampsia (4.18%) and 34 developed eclampsia (0.11%). The incidence of preeclampsia (excluding patients progressing to eclampsia) did not differ between the monsoon and the dry season (316/7346 in monsoon [4.3%] vs. 922/22216 in the dry season [4.15%], p = 0.5). The incidence of eclampsia was significantly higher in the monsoon (0.2% vs. 0.08%, p = 0.01) as compared to the dry season (Table 1).

**Table 1:** Comparison of preeclampsia, eclampsia and meteorological parameters between monsoon and dry season.

	June to August (Monsoon)	September to May (Dry)	Significance (p value)
Total deliveries	7346	22216	-
Total Preeclampsia (includes eclamptics)	331	941	0.5
Eclampsia	15	19	0.01
Maximum temperature	30.7°C	32.3°C	0.01
Morning relative humidity	85 %	70 %	0.0008
Morning barometric pressure	1005 mb	1012 mb	< 0.0001
Monthly rainfall	504.9 mm	0.3 mm	0.0002

There was a significant meteorological difference between the monsoon and rest of the year (dry season). By definition, monsoon received significantly higher monthly average rainfall when compared with the dry season i.e. rest of the year (median 504.9 mm vs. 0.3 mm respectively, p = 0.0002). The median maximum temperature in monsoon was 30.7°C and was significantly lower than during the rest of the year (32.3°C, p = 0.01). The median relative humidity in monsoon was 85% and was significantly higher than during the rest of the year (70%, p = 0.0008). The median barometric pressure) in monsoon was 1005 mb and was significantly lower than the rest of the year (1012 mb, p < 0.0001).

**Figure 1:**
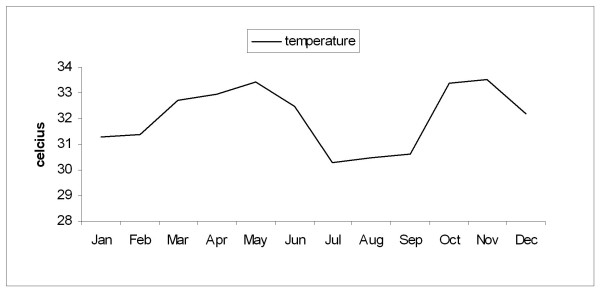
Graph showing the variation in the monthly average maximum temperature during the study period.

**Figure 2:**
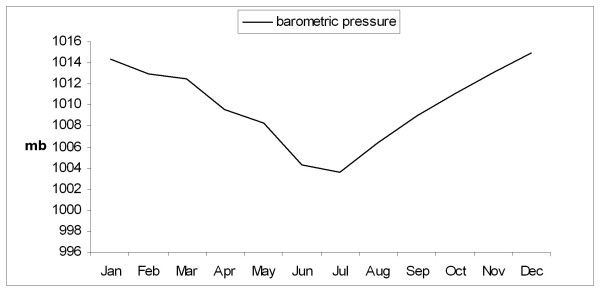
Graph showing the variation in the monthly average morning barometric pressure during the study period.

**Figure 3:**
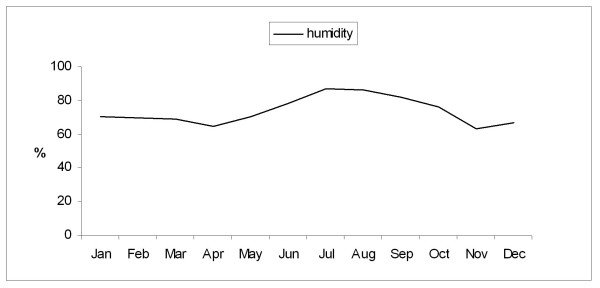
Graph showing the variation in the monthly average morning relative humidity during the study period.

**Figure 4:**
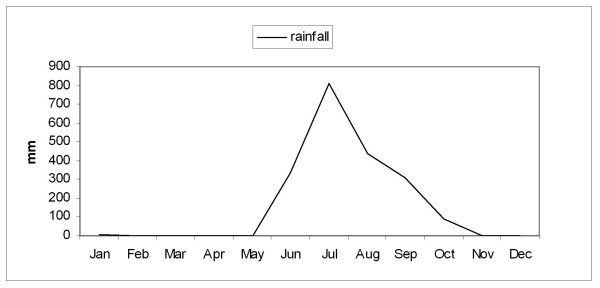
Graph showing the variation in the monthly average rainfall during the study period.

During the rest of the year (dry season, September to May) the maximum temperature, morning barometric pressure, and the relative humidity remained very stable with a mean ± SD of 32.4 ± 1.1 C, 1012 ± 2.5 mb, and 71.3 ± 8 % respectively. Figures 1, 2, 3, 4 depict the sharp changes in meteorological parameters, which occur during the monsoon season.

## Discussion

Available literature on the seasonal variation in the incidence of preeclampsia and eclampsia is much divided in its conclusion [1-13]. Preeclampsia and eclampsia are major obstetric complications with unclear aetiologies. Understanding the exact association with different weather patterns may help us in understanding what factors may be involved in triggering these events.

We evaluated a large study population over a three-year period in a large maternity centre in Mumbai for the incidence of preeclampsia and eclampsia. Preeclampsia was defined by the presence of hypertension (systolic BP ≥ 140 mm Hg or diastolic BP ≥ 90 mm Hg in a women who were previously normotensive prior) and proteinuria after 20 weeks of gestation. Eclampsia was diagnosed by the presence of seizures in addition to preeclampsia, in the absence of any other obvious cause for seizures. All patients with the diagnosis of severe preeclampsia were closely monitored, treated with magnesium sulphate infusion and delivered as soon as possible. Mild preeclampsia was treated expectantly.

Our study sample consisted of patients treated at a large maternity centre in Mumbai dealing with over 9500 deliveries a year. Our hospital treats only pre-registered patients, who have received antenatal care at our centre. All emergency un-booked referrals are taken by another state-run teaching hospital, which is next door to our centre. In effect our data is not biased by variation of referred cases as we deal with a large stable local population base. Also as our population base is local no significant delays in transporting patients to the hospital were anticipated.

Our data shows a significant increase in the incidence of eclampsia in the monsoon season, with no such change in the incidence of preeclampsia. It is widely understood that preeclampsia and eclampsia are progressive manifestations of the same patho-physiological spectrum. Our study shows that the meteorological factors had no influence on the incidence of preeclampsia. In contrast, lower temperature, higher rainfall and humidity and lower barometric pressure were related to the triggering of seizures in patients primed with preeclampsia. This is similar to other studies associating eclampsia with lower temperatures [3-5] and increased humidity and lower barometric pressures [6].

Evidence suggests that dehydration protects the brain from convulsions [14]. The warmer temperature in the dry season causes significant insensible fluid loss [15]. Westerterp et al have finely demonstrated that in women, physical activity-adjusted values of water loss were higher, especially in summer [16]. Such season related mild dehydration may play a protective role in eclampsia.

In contrast, over-hydration and hyponatremia is well known to be associated with triggering of seizures [17,18]. Hyponatremia causes direct influx of fluid into neurons causing them to swell and become more susceptible to injury and excitation. This was shown in pregnant women where decreasing serum osmolality was directly related to increasing seizure frequency [5]. Monsoon season has a direct effect on human fluid balance although the exact mechanism is unclear [19]. Chakrapani and colleagues have confirmed that the incidence of hyponatremia in hospital patients is significantly higher in the months of June to August (monsoon season) with a strong correlation to the amount of rainfall [19]. This study was done in the coastal city of Mangalore, about 500 miles south of Mumbai, having very similar climatic conditions.

In our study, we have established a significant association of eclampsia to monsoon season, which may be explained by these effects.

Contrary to the above explanation, there is some suggestion that plasma volume expands in summer when compared to winter months [20]. It is not clear if this is associated with changes in plasma osmolality. Also, this phenomenon has not been studied in tropical monsoon region. In our study temperatures during monsoon season remained high (30.7°C) although cooler than the rest of the year (32.3°C). Whether such changes in plasma volume occur in these climatic conditions is not known. The effect of seasonal variation on fluid balance, plasma volume and osmolality is poorly understood.

Our study has identified a pattern in the incidence of eclampsia, which probably is related to the climatic conditions in monsoon season. The exact mechanism by which meteorological parameters affect the patho-physiology of eclampsia is beyond the scope of our study. Further studies are needed to explore the exact mechanisms involved.

## Conclusion

There is a significant association between climatic factors and the occurrence of eclampsia, which is not seen in preeclampsia. Lower temperature, higher humidity and lower barometric pressure are linked to eclampsia. Exploring this association will help us to gain further insight into the pathophysiology of this condition.

## Competing interests

The author(s) declare that they have no competing interests.

## Pre-publication history

The pre-publication history for this paper can be accessed here:


